# Green Tea Catechin-Inactivated Viral Vaccine Platform

**DOI:** 10.3389/fmicb.2017.02469

**Published:** 2017-12-12

**Authors:** Yun H. Lee, Yo H. Jang, Young H. Byun, Yucheol Cheong, Paul Kim, Young J. Lee, Yoon J. Lee, Je M. Sung, Ahyun Son, Hye M. Lee, Jinhee Lee, Seung W. Yang, Jae-Min Song, Baik L. Seong

**Affiliations:** ^1^Department of Biotechnology, College of Life Science and Biotechnology, Yonsei University, Seoul, South Korea; ^2^Department of Global Medical Science, Health and Wellness College, Sungshin Women’s University, Seoul, South Korea; ^3^Vaccine Translational Research Center, Yonsei University, Seoul, South Korea

**Keywords:** influenza virus, green tea, catechins, inactivated vaccine, formalin

## Abstract

Traditionally, chemical agents such as formalin (FA) and β-propiolactone (BPL) have long been used for the preparation of inactivated vaccines or toxoids. It has been shown that FA extensively modifies vaccine antigens and thus affects immunogenicity profiles, sometimes compromising the protective efficacy of the vaccines or even exacerbating the disease upon infection. In this study, we show that natural catechins from green tea extracts (GT) can be used as an inactivating agent to prepare inactivated viral vaccines. GT treatment resulted in complete and irreversible inactivation of influenza virus as well as dengue virus. In contrast to FA that reacted extensively with multiple amino acids including lysine, a major anchor residue for epitope binding to MHC molecules, GT catechin epigallocatechin-3-gallate (EGCG) crosslinked primarily with cysteine residues and thus preserved the major epitopes of the influenza hemagglutinin. In a mouse model, vaccination with GT-inactivated influenza virus (GTi virus) elicited higher levels of viral neutralizing antibodies than FA-inactivated virus (FAi virus). The vaccination completely protected the mice from a lethal challenge and restricted the challenge viral replication in the lungs. Of note, the quality of antibody responses of GTi virus was superior to that with FAi virus, in terms of the magnitude of antibody titer, cross-reactivity to hetero-subtypes of influenza viruses, and the avidity to viral antigens. As the first report of using non-toxic natural compounds for the preparation of inactivated viral vaccines, the present results could be translated into a clinically relevant vaccine platform with improved efficacy, safety, productivity, and public acceptance.

## Introduction

Vaccination remains the most cost-effective means to control infectious diseases (Anderson and May, 1982). The traditional vaccination paradigm comprises three basic steps: isolation of infectious agents, inactivation, and injection into the human body to elicit protective immune responses. Advances in reverse genetics or structural vaccinology provide technical platforms for the development of new vaccines with the desired level of efficacy and safety, including virus-like particles (VLPs), vectored vaccines, and live attenuated vaccines (Sette and Rappuoli, 2010). However, most licensed viral vaccines have been produced by chemical inactivation of the viruses to eliminate the infectivity and to ensure vaccine safety. First identified as an inactivating agent for bacterial toxins, formalin (FA) has long been used to prepare a wide variety of inactivated bacterial or viral vaccines (Murdin et al., 1996; Leppla et al., 2002). Alternatively, other chemical agents such as β-propiolactone (BPL) or hydrogen peroxide can be used to eliminate viral infectivity (Logrippo and Hartman, 1955; Amanna et al., 2012). However, these chemicals must be removed by further purification to minimize potential side effects, sometimes at the expense of the productivity and the quality of the final vaccine. By crosslinking amino acids and proteinaceous materials, FA often alters the immunogenicity profiles of vaccines, occasionally exacerbating disease after vaccination, as observed in the cases of respiratory syncytial virus (RSV) and measles vaccines (Fulginiti et al., 1967; Kim et al., 1969; Muralidharan et al., 2017). In addition, side effects or autoimmune diseases following vaccination have long been observed, leading to anti-vaccine movements that jeopardize trust in vaccination as the most cost-effective means of reducing the medical burden of infectious diseases (Wraith et al., 2003; Bonhoeffer and Heininger, 2007). Clearly, there remains a need for alternative means to prepare inactivated vaccines with improved efficacy, safety, and public acceptance.

Green tea (*Camellia sinensis*) (GT) is one of the most popular beverages worldwide and is associated with many health benefits. Several lines of evidence indicate that polyphenolic catechins, most notably epigallocatechin-3-gallate (EGCG) (Supplementary Figure S1), exhibit antioxidative, antimicrobial, antitumor, and other beneficial activities (Toda et al., 1992; Valcic et al., 1999; Tachibana et al., 2004). It has also been demonstrated that EGCG exhibits broad-spectrum antiviral efficacy against diverse families of viruses, including Retroviridae, Orthomyxoviridae, Flaviviridae, Herpesviridae, Hepadnaviridae, Adenoviridae, and Picornaviridae, with different modes of actions depending on the virus (Steinmann et al., 2013). A common antiviral mechanism of EGCG stems from EGCG binding to viral surface proteins (Steinmann et al., 2013), which results in the alteration of the morphological and physical properties of the virions and inhibition of viral entry into cells, as observed for influenza virus (Nakayama et al., 1993; Imanishi et al., 2002; Song et al., 2005; Kim et al., 2013) and herpes simplex virus (Isaacs et al., 2008, 2011). Recent studies have demonstrated that EGCG forms a covalent bond with a cysteinyl thiol residue in a protein through an autoxidation mechanism, where intermolecular crosslinking between two proteins is also possible (Ishii et al., 2008; Chen et al., 2011). It is therefore reasonable to assume that the same crosslinking reaction occurs between EGCG and the viral proteins described above.

In this study, we examined whether the GT catechins could be used as a novel inactivating agent to prepare viral vaccines. Influenza virus was chosen for the development of a prototype vaccine because of the considerable medical burden associated with annual influenza epidemics and occasional pandemics worldwide (Nicholson et al., 2003). The potency of GT catechin-mediated influenza viral inactivation was investigated in detail. The GT-inactivated (GTi) influenza viruses elicited robust protective immune responses in a mouse model. In contrast to FA that extensively modifies immunological epitopes, GT preserved the major epitopes of influenza hemagglutinin, and the GTi virus resulted in significantly higher antibody titers and enhanced antibody avidity than FAi virus. As the first report of the use of non-toxic natural compounds to prepare inactivated vaccines, the present results can be translated into a clinically relevant vaccine platform for vaccines with improved efficacy, safety, productivity, and public acceptance.

## Materials and Methods

### Green Tea Extract and Chemical Agents

Green tee extract (GT) was prepared as described previously (Song et al., 2005). Breifly, green tea leaves were infused with 75°C distilled water in the ratio of 1:7 (w/w). After 20 min of infusion, the tea extract was quickly separated from the tea leaves by filtration and the tea extract was freeze-dried for further tests. The GT were analyzed for their composition by C18 reverse phase column chromatography (elution with 22% THF at the flow of 1 ml/min). The GT was composed of caffeine (5.48%), gallic acid (0.22%), GC (1.95%), EGC (10.22%), catechin (0.35%), EGCG (9.11%), EC (2.51%), and GCG (0.88%), as determined by HPLC. A GT solution was prepared by adding water to the GT powder and filtering it through a 0.2 μm syringe filter. Purified EGCG (98%, powder) was purchased from Changsha Sunfull Biotech (China). Formalin was purchased from Sigma–Aldrich (cat. no. 252549), and oseltamivir (i.e., Tamiflu^®^) was obtained from the International Vaccine Institute (Seoul, South Korea).

### Influenza Viral Proteins

The hemagglutinin (HA) and nucleoprotein (NP) proteins of A/Puerto Rico/8/1934 (H1N1) influenza viruses were expressed and purified, as described previously (Jang et al., 2014). The HA proteins were fused with *Escherichia coli* lysyl tRNA synthetase (LysRS) or rabbit RNA-binding domain (rRBD) for soluble expression in *E. coli* host, BL21star (DE3) pLysS (Invitrogen). The NP proteins were expressed in soluble form without fusion in the same *E. coli* host. The expressed proteins were purified by nickel affinity chromatography using HiTrap chelating HP column (GE Healthcare Life Sciences). LysRS-HA and rRBD-HA proteins were treated with TEV protease (AcTEV, Invitrogen) to separate the fusion partners from the HA proteins. The digested proteins (0.1 mg/mL) were incubated with various concentrations of GT (0–1,000 μg) for 6 h and then subjected to SDS-PAGE to monitor mobility changes of the proteins. Seven different recombinant HA proteins expressed in baculovirus-insect cells were purchased from Sino Biologicals (China).

### Mass Spectrometry Analysis

The HA proteins incubated with EGCG were subjected to SDS-PAGE and analyzed by liquid chromatography-MS/MS. Proteins were identified using a Q-Exactive mass spectrometer (Thermo Fisher Scientific) coupled with an Easy-nLC system (Thermo Fisher Scientific). The HA epitope peptides incubated with FA or EGCG were loaded into the heated electrospray ionization (HESI) source and measured using a selected ion-monitoring (SIM) method on a Q Exactive Hybrid Quadrupole-Orbitrap mass spectrometer (Thermo Fisher Scientific). The acquisition method consisted of two scan events, full MS and SIM. Then, respective scan parameters were set in the Tune software (Thermo Fisher Scientific). The scan type was full MS-Data dependent MS/MS. For direct infusion-SIM with HESI source, samples were loaded in a 250 μl Hamilton syringe, injected by a syringe pump with a flow rate of 3 μl min^-1^ into the HESI source and measured for 0.5 min with a SIM method on a Q Exactive Hybrid Quadrupole-Orbitrap mass spectrometer. For ionization, a spray voltage of 3.6 kV and capillary temperature of 320°C was used and sheath gas flow rate was set to 6 units. The acquisition was monitored from *m/z* 300–2000, with a resolution of 70,000 (at *m/z* 200), a maximum injection time of 200 ms and an automatic gain control value of 3e6. For direct infusion-MS/MS with HESI source, samples were injected into the mass spectrometer and ionized as described above. The following scan parameters were set in the Tune software (Thermo Scientific). The scan type was Full MS-Data dependent MS/MS and in the scan range the center *m/z* was set to the *m/z* of interest with an isolation window of 2 *m/z*. The isolated *m/z* was fragmented with a normalized collision energy of 27 in the higher-energy collisional induced dissociation cell and fragment spectra were monitored from *m/z* 200–2000, with an orbitrap resolution of 70,000 (at *m/z* 200).

### Mouse Experiments

Animal study was carried out in strict accordance with the recommendations of the Ministry of Food and Drug Safety (MFDS) of Korea. Mouse studies were reviewed and approved by the Institutional Animal Care and Use Committee (IACUC) of the Yonsei Laboratory Animal Research Center (YLARC) (201603-418-02). Six-week-old female balb/c mice were used to evaluate the safety, immunogenicity, and protective efficacy of FAi virus and GTi virus. For prime vaccination, mice (*n* = 5) were inoculated with the different doses of the inactivated viruses or PBS via intramuscular (IM) or intraperitoneal (IP) route, and boosting vaccinations were carried out 2 weeks after the prime vaccination with the same vaccination doses. Sera were taken from the vaccinated mice by retro-orbital bleeding method at every 2 weeks after the prime vaccination for antibody response analysis. The mice were challenged by intranasal infection with ten mouse lethal dose (10^4^ PFU) of PR8 virus at 4 weeks after the boosting vaccination, and the mice were monitored daily for clinical signs and mortality. Separate groups of mice (*n* = 4) were sacrificed at 2, 4, and 6 days after the challenge for the measurement of the viral titers in the lung and nasal turbinate. All animal vaccination and challenge experiments were conducted under anesthesia. Mice that lost weight greater than 25% of initial weight were considered non-viable and euthanized, according to the animal experiment protocol approved by the IACUC.

### Measurement of HI and NT Antibodies

For hemagglutination inhibition (HI) assay, clarified sera were pretreated with receptor destroying enzyme to minimize non-specific inhibition and then heat-inactivated for 30 min at 56°C. Twofold serial dilutions of sera (25 μL) were incubated with an equal volume of influenza viruses (4 HAU) for 1 h at 37°C. Then, 50 μL of 1% cRBCs was added to each well and incubated for 1 h at 4°C. HI antibody titers were expressed as the highest serum dilution that completely inhibited hemaggutination. For neutralization (NT) assay, twofold serial dilutions of the sera were incubated with 100 PFU of influenza virus for 1 h at 37°C. The mixtures were subjected to plaque assay on MDCK cells, and the plates were maintained at 37°C in 5% CO_2_ until plaques became visible. For plaque assay, MDCK cells in 12-well plates were infected with 10-fold serial dilutions of virus and incubated for 1 h at room temperature (RT). The inocula were removed, and the cells were overlaid with medium containing 1% low-melting agarose, Dulbecco’s modified Eagle medium, and 10 μg/mL trypsin. Viral plaques were counted after 3 days of incubation at 37°C in a CO_2_ incubator. NT antibody titers were defined as the dilution that corresponded to a 50% plaque reduction as compared to those in the control.

### Measurement of Relative Avidity of Antibodies by ELISA

ELISA with the inclusion of urea wash procedure was performed to measure the avidity of vaccination-induced antibodies to viral surface antigens, as described previously (Bachmann et al., 1997; Fleury et al., 1999; Polack et al., 2003; Delgado et al., 2009). 96-well plates were coated with 10^4^ PFU of PR8 virus, and the wells were blocked by 1% BSA for 1 h at RT. After washing, twofold serial dilutions of sera were added to the plates, followed by incubation for 1 h at RT. After washing, 100 μL of distilled water (DW) or 7–9 M urea dissolved in DW were added to each well and incubated for 30 min for the dissociation of antibodies from the viruses. The urea was removed by washings and HRP-conjugated goat anti-mouse IgG antibody (Bethyl Laboratories) was added to the wells and incubated for 1 h. After washing, TMB substrate solution was added to the wells and incubated for 30 min. The colorimetric reaction was stopped by the addition of 1 M H_2_SO_4_, and the absorbance was read at 450 nm on an ELISA reader.

### Statistical Analysis

All data are expressed as the mean for each group, and error bars indicate standard deviation (SD). To determine statistical significance, a Student’s *t*-test was used when comparing two different groups. *P* < 0.05 was considered statistically significant.

## Results

### Inactivation of Influenza Virus by GT Treatment

**FIGURE 1:**
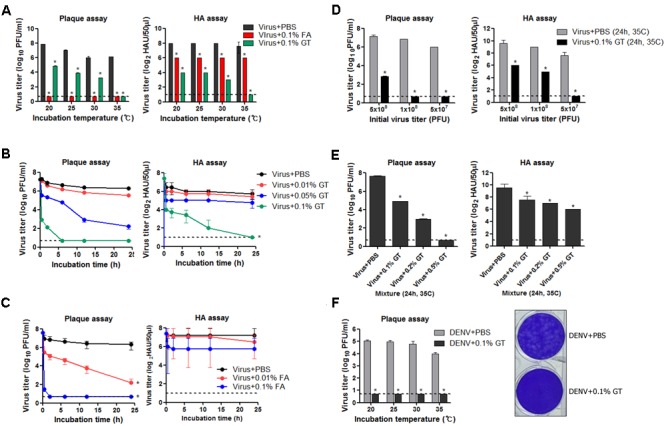
Inactivation of influenza virus by incubation with GT. **(A)** Effect of incubation temperature on the activity of GT. GT (1 mg/mL, 0.1% w/v) or FA (0.1% v/v) was mixed with 5 × 10^7^ PFU of PR8 virus, and the mixtures were incubated for 24 h at four different temperatures. After the incubation, viral titers in the mixtures were estimated by plaque assay and hemagglutination assay. **(B)** Time-course of viral inactivation by GT. PR8 virus (5 × 10^7^ PFU) was incubated with three different concentrations of GT (0.01–0.1%) at 35°C and the mixtures were taken at various time points to measure viral titers by plaque assay and hemagglutination assay. **(C)** Time-course of viral inactivation by FA. PR8 virus (5 × 10^7^ PFU) was incubated with two different concentrations of FA (0.01% and 0.1%) at 35°C, and then the mixtures were harvested at various time points to measure viral titers by plaque assay and hemagglutination assay. **(D)** Inactivation potency of GT. Three different titers of PR8 virus were incubated with 0.1% GT for 24 h at 35°C. Viral titers of the mixtures were determined by plaque assay and hemagglutination assay. **(E)** Inactivation of 5 × 10^8^ PFU of PR8 virus by GT treatment. 5 × 10^8^ PFU of PR8 virus were mixed with three different concentrations of GT (0.1–0.5%) and the mixtures were incubated for 24 h at 35°C. After the incubation, viral titers in the mixtures were estimated by plaque assay and hemagglutination assay. **(F)** Inactivation of DENV by GT. 1 × 10^6^ PFU of dengue virus (DenKor-01) was mixed with 0.1% GT and incubated for 24 h at four different temperatures. After the incubation, viral titers in the mixtures were measured by plaque assay on Vero cells. A represantative plaque assay image of DENV treated with PBS or 0.1% GT is shown. Data are the means of three independent experiments. Dashed lines indicate the detection limits of plaque assay and hemagglutination assay. ^∗^*P* < 0.05 when comparing antibody titers between two different groups.

To examine the virucidal effect of GT or FA against influenza viruses through direct contact, 5 × 10^7^ plaque-forming units (PFUs) of influenza A virus (A/Puerto Rico/8/1934 H1N1) (PR8) virus dissolved in 1 mL of PBS was mixed with 1 mg/mL of GT (0.1% w/v) or FA (0.1% v/v) and incubated for 24 h at various temperatures. After the incubation, viral titers in the mixtures were measured by plaque assay and hemagglutination assay to estimate the residual plaque-forming ability and HA activity, respectively. FA completely inactivated viral infectivity, as no viral plaques were detected after the incubation at all the temperatures tested (**Figure 1A**, left). Incubation with GT at 20–30°C resulted in a ∼1,000-fold decrease in PFU titers, whereas no plaques were detected (<10^7^ reduction) after incubation at 35°C. Incubation with FA resulted in fourfold decrease in HA units (HAU), irrespective of incubation temperatures (**Figure 1A**, right). Incubation with GT at 20–30°C resulted in a 16- to 30-fold decrease in HAU titers compared to that of the PBS-treated control, and incubation at 35°C completely removed the HA activity. The time-course of viral inactivation showed that GT treatment decreased the viral PFU and HAU titers in a time- and dose-dependent manner (**Figure 1B**). 0.1% GT eliminated both viral plaque-forming ability and HA activity after incubation for 24 h. Notably, the infectivity was eliminated as early as 6 h after incubation with 0.1% GT, demonstrating the potent inactivating activity of GT. Following GT treatment, HA activity decreased more slowly than infectivity, suggesting that GT primarily affects the HA conformation in a non-fusogenic state and that GT affects receptor-binding activity with prolonged treatment. For comparison, influenza viruses were treated with FA and viral inactivation was monitored using the same protocols as for GT treatment. Treatment with 0.01% (v/v) FA resulted in 5 log_10_ reduction in viral PFU titers after 24 h, whereas 0.1% FA resulted in <10^6^ reduction within 2 h (**Figure 1C**). Despite the loss of viral infectivity, a substantial level of HA activity was maintained during incubation with FA. The potency of inactivation by GT was evaluated by incubating the various titers of viruses with a fixed amount of GT. 0.1% GT was able to completely inactivate 5 × 10^7^ and 1 × 10^8^ viral PFU (i.e., a > 10^7^-fold reduction). With an initial amount of 5 × 10^8^ viral PFU, partial inactivation (>10^5^-fold reduction) was observed (**Figure 1D**), whereas an increase in GT concentration to 0.5% led to complete viral inactivation (∼10^8^-fold reduction in viral titers) (**Figure 1E**). To test whether GT could inactivate other virus as well, 1 × 10^6^ PFU of dengue virus (Dengue virus type 1 isolate DenKor-01) was incubated with 0.1% GT or PBS for 24 h at 20–35°C, and residual DENV titers were estimated by plaque assay on Vero cells. Incubation with GT completely removed the infectivity of DENV, as no viral plaques were detected at all the temperatures tested (**Figure 1F**). Distinct to influenza virus, DENV is the member of the family of *Flaviviridae*, and, thus, the results suggest that viral inactivation by GT could be applicable into diverse virus families.

### GT Catechins Crosslink with Influenza Viral Proteins

**FIGURE 2:**
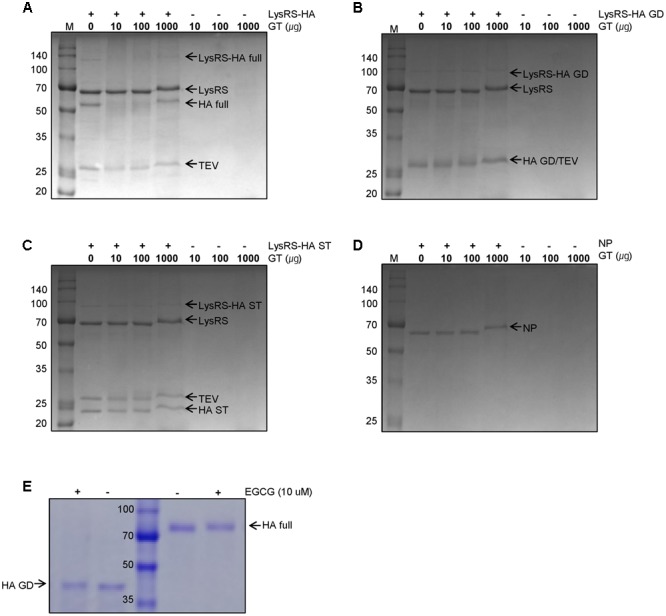
Modifications of influenza viral proteins by GT treatment. **(A–C)** Modification of influenza HA proteins by GT. *E. coli*-expressed LysRS-HA full-length **(A)**, globular domain (GD) **(B)**, and stalk (ST) **(C)** proteins were digested by TEV protease before GT treatment. The digested proteins were treated with the various concentrations of GT (0–1,000 ug) for 6 h at 35°C and were subjected to SDS–PAGE. **(D)** Modifications of influenza NP proteins by GT treatment. *E. coli*-expressed NP proteins were incubated with GT (10–1000 μg) for 6 h at 35°C and their molecular weight change was examined by SDS-PAGE. **(E)** Modification of HA proteins by EGCG treatment. *E.coli*-expressed rRBD-HA GD and HEK293 cell-expressed HA full-length proteins were incubated with 10 μM of EGCG for 2 h at RT and their mobility changes were analyzed by SDS-PAGE.

Based on previous observations that EGCG, a major GT catechin, induced protein crosslinking (Ishii et al., 2008; Chen et al., 2011), we examined whether GT catechins were covalently conjugated with influenza viral proteins during inactivation. For this purpose, influenza HA full-length, globular domain (GD), and stalk (ST), were expressed in *E. coli* as soluble fusion proteins (Jang et al., 2014). These proteins strongly reacted with antisera to PR8 virus, suggesting that the proteins have biologically relevant conformations (Supplementary Figure S2). The NP was also expressed as soluble proteins. The HA and NP proteins were incubated with various concentrations of GT, and their mobility shifts were analyzed by SDS-PAGE. Incubation with GT resulted in a slight but distinct shift to a higher molecular weight for both HA and NP proteins (**Figures 2A–D**) that was indicative of GT catechin-based covalent modification of the viral proteins. A similar band shift to a higher molecular weight was also observed when pure EGCG were incubated with the HA GD or full-length proteins (**Figure 2E**). The HA proteins incubated with EGCG were analyzed by mass spectrometry (MS) to determine the identity of the modified amino acid residues and the extent of modification. The percentage modification of each protein or peptide was calculated from the MS data and summarized in **Table 1**. Mass differences showed that the major crosslinking of dehydroepigallocatechin (a modified form of EGCG) to the HA occurred at cysteine-152 (51.9%), with minor modifications at cysteine-319 and tyrosine-222 (0.7%) (**Table 1** and Supplementary Figure S3A). These results were consistent with previous studies showing crosslinking between the hydroxyl group of catechins and the thiol group of cysteine residues in model peptides (Ishii et al., 2008; Chen et al., 2011). It should be noted that among 12 cysteine residues in the entire HA protein, cysteine-152, which is located close to the receptor (sialic acid) binding site (RBS) of the HA, was the most predominant residue modified by EGCG (Supplementary Figure S3B). While FA completely eliminated viral infectivity but preserved the HA activity of influenza virus, GT treatment resulted in the loss of both infectivity and HA activity (**Figures 1A,B**). In parallel, three linear HA epitope peptides, Sa (156–167), Sb (187–198), and Cb (77–82) (Caton et al., 1982), were treated with either FA or EGCG, and their modifications were also analyzed by MS. The FA-treated epitope peptides showed various mass changes relative to the untreated control (Supplementary Figure S4), reflecting nucleophilic attack followed by dehydration (Metz et al., 2004). Following FA treatment, more than 60% of the epitope peptides harbored covalent modifications at multiple amino acids including lysine, tyrosine, asparagine, and tryptophan residues (**Table 1** and Supplementary Figures S4A–C). By contrast, EGCG-treated epitope peptides were modified at limited residues including tyrosine at a much lower frequency (11–14%). The results show that EGCG primarily reacts with cysteine residues in the HA, resulting in much fewer covalent modifications to the HA epitopes than FA.

**Table 1:** Covalent modification of influenza HA protein and epitopes by EGCG.

Modifier	Target	Modified region^a^	% Modification^b^
EGCG	HA protein	SWPNHNTNGVTAA**C_152_**SHEGKSSFYR	51.9
		EQQNLYQNENAYVSVVTSN**Y_222_**NR	0.7
		NIHPVTIGE**C_319_**PK	Not determined
	HA epitope	EKEGS**Y**PKLKNS (Sa)	11.1
		NSKEQQNLYQNE (Sb)	0
		DPLLPVRSWSYI (Cb)	14.1
Formaldehyde	HA epitope	E**K**EGS**Y**P**K**LKNS (Sa)	63.2
		**N**S**K**EQQNL**Y**QNE (Sb)	65.6
		DPLLPVRS**W**SYI (Cb)	68.7

*^a^Modified amino acid residues are shown in bold and underlined. ^b^% Modification was obtained from mass spectrometry data shown in Supplementary Figures S2, S3.*

### Irreversible Inactivation of Influenza Virus with GT Treatment

**FIGURE 3:**
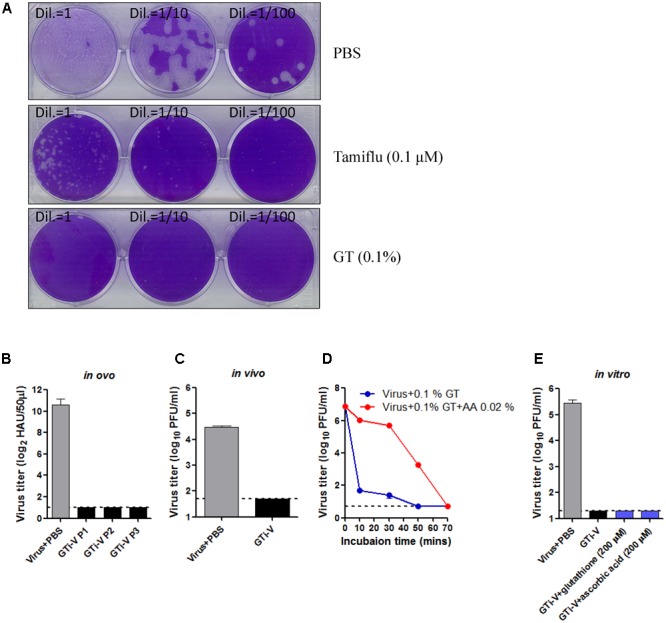
Irreversible inactivation of influenza virus by GT treatment. **(A)** Inactivation of the plaque-forming ability by GT treatment. 1,000 PFU of PR8 virus was incubated with PBS, Tamiflu (0.1 μM), or GT (0.01%) for 6 h at RT. A 10-fold dilutions of the mixtures were then subjected to plaque assay on MDCK cells. The plates were incubated at 37°C for a week before fixation and staining. **(B–E)** Validation of the irreversible inactivation of influenza virus by GT treatment. **(B)** Validation *in ovo*. PR8 virus (5 × 10^7^ PFU) was incubated with PBS or GT (0.1%), and the mixtures were injected into embryonated chicken eggs and incubated for 3 days at 37°C. Allantoic fluids were harvested at each passage for viral titration by hemagglutination assay. Data are the means of three independent experiments. **(C)** Validation *in vivo*. PR8 virus (2.5 × 10^6^ PFU) was incubated with PBS or GT (0.1%), and the mixture was intranasally injected into mice (*n* = 3). Four days later, the lungs were taken from the mice for viral titration by plaque assay. **(D)** Time-course of viral inactivation by GT in the presence of ascorbic acid. PR8 virus (5 × 10^7^ PFU) was incubated with GT (0.1%) and ascorbic acid (0.02%) at 25°C, and the mixtures were harvested at various time points for viral titration by plaque assay. Data are the means of three independent experiments. **(E)** Validation *in vitro*. PR8 virus (1 × 10^6^ PFU) was incubated with PBS or GT (0.1%) for 2 h at 35°C. After viral inactivation, 200 μM glutathione or ascorbic acid was added to the mixtures and incubated for 2 h at 37°C. The final mixtures were subjected to plaque assay for viral titration. Data are the means of three independent experiments.

In addition to the extent of viral inactivation, irreversibility is a prerequisite for a promising inactivating agent. To test this, influenza viruses were incubated with GT or Tamiflu, a well-known competitive inhibitor of viral neuraminidase (Gubareva et al., 2000), prior to a plaque assay onto MDCK cells. With Tamiflu treatment, visible plaques were observed after incubation for a week, although plaques were much smaller than the PBS-treated control (**Figure 3A**), consistent with the reversible nature of a competitive inhibitor. By contrast, GT treatment completely abrogated viral infectivity, without the formation of any plaques even after prolonged incubation, suggesting irreversible inactivation. To further validate the elimination of viral infectivity, GT-treated viruses were inoculated and passaged three times in embryonated chicken eggs, and the viral titers at each passage were measured by hemagglutination assay. HA activity was not observed at any of the passages, suggesting complete and irreversible inactivation (**Figure 3B**). Viral inactivation was further substantiated in an animal model. Mice were intranasally (IN) inoculated with GT-treated PR8 virus (2.5 × 10^6^ PFU), and the lung viral titers were measured at 4 days post-infection. No residual infectivity of GT-treated viruses was detected, in contrast to persistent viral replication (2.8 × 10^4^ PFU/mL) of PBS-treated viruses (**Figure 3C**). Viral inactivation rate was significantly decreased in the presence of ascorbic acid, suggesting that a reducing agent dampened the oxidative conjugation of GT to the virions (**Figure 3D**). However, when reducing agents were added after inactivation, viral plaques were not detected, even after prolonged incubation with high concentrations of the reducing agents (**Figure 3E**). These results suggest that the oxidative damage to virions cannot be repaired, indicating irreversible inactivation of viral infectivity.

### Neutralizing Antibodies Induced by GTi Influenza Viruses

**FIGURE 4:**
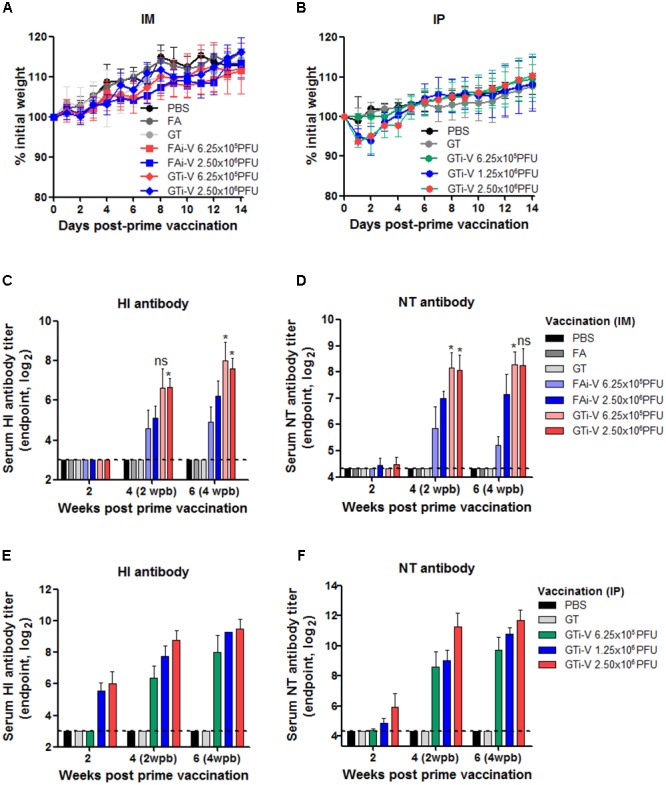
Neutralizing antibody responses elicited by GTi influenza viruses. **(A,B)** Vaccination of mice with FAi or GTi virus. Mice (*n* = 4 or 5) were vaccinated with FAi or GTi virus with an interval of 2 weeks, via IM **(A)** or (IP) **(B)** route. PBS, FA, and GT were used as vehicle controls. Weight changes in the vaccinated mice were monitored daily. **(C,D)** Neutralizing antibody titers elicited by IM vaccination. HI antibody titers **(C)** and NT antibody titers **(D)** in sera obtained at 2, 4 (2 weeks post-boost vaccination), and 6 (4 weeks post-boost vaccination) weeks after the prime vaccination are shown. **(E,F)** Neutralizing antibody titers elicited by IP vaccination. HI antibody titers **(E)** and NT antibody titers **(F)** are shown. The values are the mean of each cohort. Dashed lines indicate the detection limits of NT assay and HI assay. ^∗^*P* < 0.05 when comparing antibody titers between two different groups.

We then examined the immunogenicity of GT-inactivated (GTi) viruses in a mouse model. Mice were vaccinated with different doses of GTi virus via intramuscular (IM) or intraperitoneal (IP) route and boosted with the same doses at 2 weeks after the prime vaccination. While IM vaccination with GTi virus did not result in weight loss in the mice (**Figure 4A**), IP vaccination with higher antigen doses resulted in temporary weight loss of ∼7% (**Figure 4B**). Mice did not develop detectable levels of HI antibodies at 2 weeks after IM vaccination with FAi or GTi viruses (**Figure 4C**). Following boost vaccination, however, HI antibody titers were substantially increased in all vaccination groups, as shown by the antibody titers in the sera obtained at 2 and 4 weeks after the boost vaccination. NT antibody titers demonstrated similar trends to the HI antibody titers. NT antibodies were rarely detectable at 2 weeks after the prime vaccination but substantially increased by boost vaccination (**Figure 4D**). Of note, GTi virus induced fourfold to eightfold higher HI and NT antibody titers than FAi virus at the same vaccination doses. IP vaccination was also highly immunogenic, developing HI and NT antibodies in a dose-dependent manner (**Figures 4E,F**). With the same vaccination doses of GTi virus, IP vaccination resulted in fourfold to eightfold higher HI and NT antibody titers than IM vaccination. The results demonstrate that GTi virus is highly immunogenic in mice, resulting in high levels of neutralizing antibodies against the wild type virus.

### Quality of Antibody Responses Elicited by GTi Influenza Virus

**FIGURE 5:**
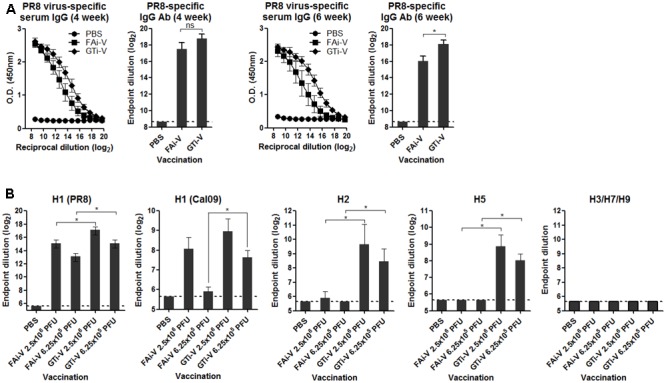
Antibody response to influenza virus and HA proteins. **(A)** Influenza virus-specific antibody responses. Twofold serial dilutions of antisera to FAi virus or GTi virus (vaccination dose; 2.5 × 10^6^ PFU) were bound to PR8 virus (10^4^ PFU/well) to measure the virus-specific IgG antibodies by ELISA. IgG antibody titers in sera obtained at 4 weeks post prime vaccination and 6 weeks post prime vaccination are shown. Antibody titers were expressed as the endpoint dilution that yielded OD_450_ greater than the mean +2 SD of PBS control group. The values are the mean of each cohort (*n* = 5). **(B)** HA-specific antibody responses. Twofold serial dilutions of antisera to FAi virus or GTi virus (vaccination dose; 6.25 × 10^5^ PFU and 2.5 × 10^6^ PFU) were bound to recombinant HA proteins to measure HA-specific IgG antibody titers by ELISA. The recombinant HA proteins tested include the homologous HA of PR8 (H1N1) and the heterologous HAs of A/California/6/2009 (H1N1), A/Canada/720/2005 (H2N2), A/Indonesia/5/2005 (H5N1), A/Sydney/5/1997 (H3N2), A/Anhui/1/2013 (H7N9), and A/Hong Kong/35820/2009 (H9N2). Antibody titers were expressed as the endpoint dilution that yielded OD_450_ greater than the mean +2 SD of the control group. The values are the mean of each cohort (*n* = 5). ^∗^*P* < 0.05 when comparing antibody titers between two different groups.

**FIGURE 6:**
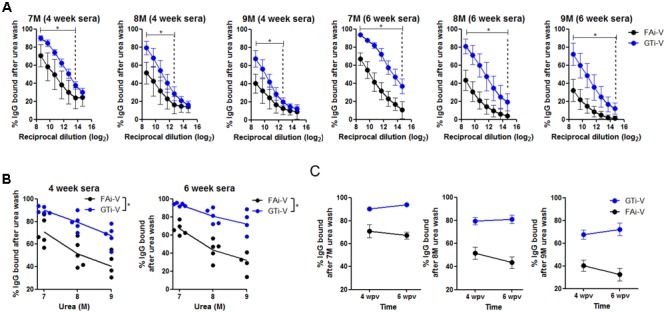
Quality of antibody responses elicited by GTi virus. **(A)** Comparison of relative avidity of antibodies elicited by GTi virus or FAi virus. 96-well plate wells were coated with PR8 viruses (10^4^ PFU/well), and twofold serial dilutions of antisera to GTi virus or FAi virus (vaccination dose; 2.5 × 10^6^ PFU) were added to the PR8 virus for binding. The wells were then washed with either distilled water (DW) or 7-9 M urea for 30 min at RT. The percentages of IgG antibodies bound after the urea washing were expressed as % OD_450_ relative to that of DW washing. The vertical dashed lines indicate the lowest serum concentrations that show statistically significant differences between vaccinations with GTi virus and FAi virus. **(B)** The trends of the antibody avidity at the highest serum concentration along urea concentrations. **(C)** Maturation of antibody avidity over time after vaccination with GTi virus. Data are the mean of each cohort (*n* = 5). ^∗^*P* < 0.05 when comparing antibody avidity between two different groups.

To measure vaccination-induced antibodies specific to influenza surface HA and NA proteins, sera were bound to PR8 virus and specific IgG antibody titers were estimated by ELISA. With the same vaccination dose (2.5 × 10^6^ PFU), GTi virus resulted in twofold to fourfold higher levels of anti-influenza specific IgG antibodies in antisera obtained at four and 6 weeks after prime vaccination, as compared to FAi virus (**Figure 5A**). HA-specific antibody titers were also measured against the homologous or heterologous HA proteins. Against the homologous PR8 HA, GTi virus induced higher serum IgG antibody titers than FAi virus across different doses of vaccination (**Figure 5B**). Notably, vaccination with GTi virus resulted in significant increases in cross-reactive HA-specific antibodies against the heterologous H1, and hetero-subtypic H2, and H5 HA proteins, as compared to FAi virus (**Figure 5B**). Interestingly, even a lower dose of GTi virus (6.25 × 10^5^ PFU) elicited higher cross-reactive antibodies against the H2 and H5 HA proteins than higher dose of FAi virus (2.5 × 10^6^ PFU), showing robust ability of the GTi virus to elicit cross-reactive antibody responses. Neither vaccination with GTi virus nor FAi virus induced detectable levels of cross-reactive antibodies against H3, H7, or H9 HAs. Next, to examine the quality of the antibody response by GTi virus, we compared relative antibody avidity induced by vaccination with GTi virus or FAi virus with ELISA including urea washes, as previously described for influenza and other viruses (Bachmann et al., 1997; Fleury et al., 1999; Polack et al., 2003; Delgado et al., 2009). Serial dilutions of antisera to FAi or GTi virus were bound to PR8 virus and then washed with 7–9 M urea solution, and antibodies that remained bound after the wash were assessed by ELISA. Antisera against GTi virus obtained at four and 6 weeks after the prime vaccination showed higher avidity than antisera to FAi virus, at all the urea concentrations tested (**Figures 6A,B**). Of note, the avidity of the antibodies elicited by FAi virus was decreased from 4 to 6 weeks after prime vaccination, whereas vaccination with GTi virus resulted in improvement of antibody avidity during that period (**Figure 6C**), suggesting that the avidity maturation process was rendered more active by vaccination with GTi virus than by FAi virus. Taken together, the results suggest that GTi virus with relatively less covalent modifications in the viral antigens elicited antibody responses of higher avidity than FAi virus carrying extensive modifications.

### Protective Efficacy of GTi Influenza Viruses

**FIGURE 7:**
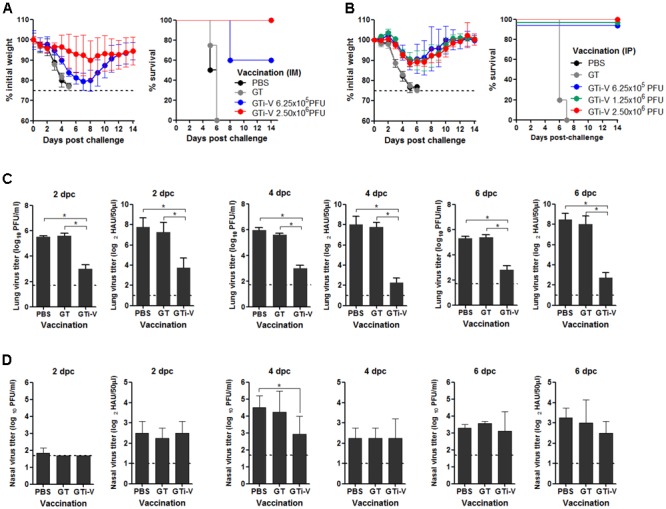
Protective efficacy of GTi influenza viruses. **(A,B)** Protection against lethal challenge with PR8 virus. Mice (*n* = 5) with IM **(A)** or IP **(B)** vaccination with different doses of GTi virus, and control mice given PBS were challenged with 10 MLD_50_ of PR8 virus at 4 weeks after boost vaccination. After the challenge, the weight changes and survival of the mice were monitored daily. Mice that lost their initial weights more than 25% were considered as non-viable and euthanized. **(C,D)** Challenge viral replication in the mouse respiratory tracts. Separate groups of mice (*n* = 4) were primed and boosted with 2.5 × 10^6^ PFU of GTi virus via IP route, and control mice were inoculated with PBS or GT. Four weeks later, the mice were challenged with 10 MLD_50_ of PR8 virus, and the lung and nasal turbinate were taken at 2, 4, or 6 days post-challenge for viral titration. The viral PFU titers and HAU titers in the lungs **(C)** and nasal turbinate **(D)** are shown. Dashed lines indicate the detection limits of the assay. ^∗^*P* < 0.05 when comparing two different groups.

To examine the protective efficacy of GTi virus, IM vaccinated mice were challenged with 10 mouse lethal dose 50 (MLD_50_) of wild type PR8 virus. The control mice that were given PBS or GT showed rapid weight loss and succumbed to death within 6 days post-challenge (**Figure 7A**). Vaccination with 6.25 × 10^5^ PFU of GTi virus provided a partial protection against the challenge, developing a weight loss of ∼20%. A fourfold increase in vaccination dose to 2.5 × 10^6^ PFU of GTi virus reduced the weight loss of the challenged mice to ∼10% and provided complete protection. IP vaccination with GTi virus also protected the mice from the lethal challenge (**Figure 7B**). Upon the challenge with 10 MLD_50_ of PR8 virus, all vaccinated mice displayed mild weight loss of ∼10% and fully recovered without death, whereas non-vaccinated control mice showed rapid weight loss and succumbed to death within 7 days post-challenge. To assess sterile immunity in the respiratory tracts, vaccinated mice were challenged with PR8 virus, and the viral titers in the lung and nasal turbinate were estimated at various time points after the challenge. Vaccination with GTi virus significantly reduced the lung viral PFU and HAU titers at 2, 4, and 6 days post-challenge, as compared to the controls receiving PBS or GT (**Figure 7C**), indicating that the vaccination efficiently restricted replication of the challenge virus in the lungs. However, the vaccination reduced the viral replication only slightly in the nasal turbinate (**Figure 7D**). Thus, the observed protection was mediated primarily by humoral rather than mucosal immune responses, which is commonly observed in other inactivated influenza vaccines administered via IM route (Ghendon, 1990; Beyer et al., 2002).

## Discussion

In this study, GT was successfully presented as an effective inactivating agent for the preparation of viral vaccines. GT was able to inactivate influenza virus as well as DENV. Considering that DENV belongs to the virus family of *Flaviviridae*, our results show the possibility that GT could be used as a universal inactivating agent for distinct virus families. While incubation with FA maintained substantial level of HA activity of the influenza virus, GT led to a potent and irreversible inactivation of both the viral infectivity and HA activity of the influenza virus. This difference between FA and GT may reflect fundamental differences in the mechanisms of inactivation involved. Considering the proximity of cysteine-152 to the receptor binding region of the HA (Supplementary Figure S3B), modification of this residue by EGCG is likely to interfere with interactions between the sialic acid and the HA. MS analysis of influenza HA proteins revealed that EGCG reacted primarily with cysteine residues (51.9%) and with tyrosine residues at extremely low rates (0.7%). Consistent with this, three linear HA epitope peptides lacking cysteine residues were modified by EGCG treatment only at tyrosine residues at a low level (11.1%). In marked contrast to EGCG, FA reacted with multiple amino acids in the epitope peptides, including lysine, tyrosine, asparagine, and tryptophan, at substantially higher rates (>60%). The known antigenic epitopes of the HA of PR8 virus carry eight lysine residues, representing 16% (8/50) of total lysine residues in the protein, whereas no cysteine residues participate in the epitopes, despite the existence of nine and three residues in the globular domain and the stalk domain of the HA, respectively (Caton et al., 1982). Thus, FA treatment is likely to result in extensive modifications in the HA epitopes. By contrast, EGCG reacted primarily with cysteine residues that were not found in the HA epitopes, leaving most epitopes unaffected during inactivation.

Better preservation of antigenic epitopes during GT-mediated inactivation of influenza viruses conferred beneficial effects on the quality of antibody responses. GTi virus induced higher levels of viral neutralizing antibodies than FAi virus in vaccinated mice, even with fourfold lower vaccination dose (**Figures 4C,D**). Furthermore, the cross-reactivity of the antibody responses by GTi virus extended into the heterosubtypic HA proteins such as H2 and H5 HA proteins, to which antisera to FAi virus barely reacted (**Figure 5B**). These results clearly indicate that vaccination with GTi virus developed stronger and broader antibody responses toward homologous, heterologous, and even hetero-subtypic influenza viruses than FAi virus. Finally, antibody responses elicited by GTi virus not only demonstrated higher avidity to PR8 virus (**Figure 6B**), but the avidity was increased over time after vaccination, in clear contrast to that of FAi virus (**Figure 6C**). Higher cross-reactive antibody titers and enhanced antibody avidity demonstrated by GTi virus could be attributable to a better preservation of epitopes in viral antigens by GT treatment. Certainly, the elucidation of precise mechanisms for superior avidity maturation shown by GTi virus merits further studies.

During the antigen processing and presentation by antigen presenting cells (APCs), epitope peptides are loaded onto MHC molecules, where the anchor residues play crucial role for preferential binding to specific pockets on the MHC molecule (Maffei and Harris, 1998). It is likely that better preservation of the epitope anchor residues can significantly improve the quality of antigen presentation and subsequent antibody responses. To examine the possibility that better preservation of epitopes by GT treatment has wide applicability to different vaccine antigens, the amino acid compositions of linear epitopes in a database and the relative importance of each amino acid for binding to human MHC molecules were examined (**Table 2**). The analysis revealed that among 20 amino acids, cysteine turned out to be the least occurring (1.13% of total residues) in the repertoire of epitopes. More importantly, cysteine neither participated as a primary nor a secondary anchor residue in peptide binding to 18 selected alleles of human MHC class-I and class-II molecules. Based on this analysis, it is reasonable to suggest that EGCG can serve as a promising inactivating agent for preparing a wide range of vaccines that elicit antibodies with higher avidity to antigens than classical FAi vaccines. The chemical reactivity of FA to proteins or peptides has been well-documented in previous studies (Wadsworth and Pangborn, 1936; Metz et al., 2004; Toews et al., 2008), in which 16 out of a total 20 amino acids were shown to be covalently modified by FA treatment (**Table 2**). Thus, purely based on chemical reactivity, FA treatment is expected to result in the extensive alteration of epitopes that may affect antigenic structure, antigen processing, and eventually, the quality of immune responses, as exemplified by poliovirus vaccine and pertussis toxoids (Ferguson et al., 1993; Ibsen, 1996). In addition, as documented for FAi RSV vaccine, concerns have been raised that FA treatment may cause an imbalance in T_H_2/T_H_1 immune responses, contributing to vaccine-associated enhancement of disease (Moghaddam et al., 2006). Moreover, an alternative inactivating agent BPL also led to extensive modification of influenza HA and NA surface proteins (8 out of 20 amino acids) (**Table 2**) (She et al., 2013). In clear contrast to these chemicals, EGCG reacted with only two amino acids, primarily cysteine and rarely tyrosine.

**Table 2:** Amino acid composition of linear epitopes and their relative importance in human MHC binding.

Amino acid	Composition ratio (%)^a^	Relative importance to MHC binding^b^		Modification^c^		
		MHC class-I	MHC class-II	FA	BPL	EGCG
Cys (C)	1.13	– (0,0)	– (0,0)	*√*		*√*
Lys (K)	5.67	+++ (7,1)	+++ (5,0)	*√*	*√*	
Tyr (Y)	4.45	+++ (6,3)	+++ (7,2)	*√*	*√*	*√*
Trp (W)	1.46	++ (1,1)	++ (2,1)	*√*		
Asn (N)	4.02	– (0,0)	++ (2,4)	*√*		
Arg (R)	5.04	+++ (3,1)	+++ (4,1)	*√*		
His (H)	2.34	– (0,0)	+ (0,1)	*√*	*√*	
Asp (D)	4.39	++ (1,0)	++ (1,1)	*√*	*√*	
Glu (E)	6.09	++ (2,0)	++ (1,0)	*√*	*√*	
Ser (S)	6.92	+ (0,1)	+ (0,1)	*√*	*√*	
Thr (T)	5.80	++ (1,1)	+ (0,1)	*√*	*√*	
Gln (Q)	4.13	– (0,0)	++ (1,2)	*√*		
Gly (G)	5.88	– (0,0)	++ (1,0)	*√*		
Pro (P)	5.60	++ (2,1)	++ (1,0)			
Ala (A)	7.65	++ (1,2)	+++ (4,3)	*√*		
Val (V)	6.56	++++ (4,6)	++++ (8.5)			
Ile (I)	5.39	+++ (3,6)	++++ (8,5)			
Leu (L)	10.35	++++ (11,5)	+++ (7,2)	*√*		
Met (M)	2.38	+++ (5,3)	+++ (5,2)		*√*	
Phe (F)	2.35	++++ (5,5)	++++ (9,3)	*√*		

*^a^MHC class-I or class-II restricted, disease-related, human B cell, and T cell linear epitopes were selected (167,965 epitopes in total) from the IEDB (http://www.iedb.org/) database. The ratio of each amino acid was the frequency at which an amino acid appeared among the 167 965 linear epitopes selected. ^b^Based on a previous report (Maffei and Harris, 1998), the relative importance of each amino acid in binding to an MHC molecule was determined based on the number of inclusion (NOI) as primary or secondary anchor residues in the 11 alleles of MHC class-I molecules and 7 alleles of MHC class-II molecules, as follows: ++++, NOI as anchor residues ≥ 10; +++, NOI as primary anchor residues ≥ 3; ++, NOI as residues ≥ 1; +, included as secondary anchor residues only; -, not included as anchor residues. For each amino acid, NOI as a primary anchor residue and NOI as a secondary anchor residue are given in parentheses. ^c^Modification of amino acid residues following FA or BPL treatment was reported in previous studies (Wadsworth and Pangborn, 1936; Metz et al., 2004; Toews et al., 2008; She et al., 2013), and modifications in response to EGCG were reported in the current and previous study (Ishii et al., 2008).*

In our previous study, effective concentration 50 (EC_50_) of GT against 500 PFU of influenza viruses was 63.8–70.5 μg/mL, and 200 μg/mL of GT was able to completely inactivate approximately 5 × 10^4^ PFU of the viruses (Song et al., 2005). In the present study, 1 mg/mL of GT (0.1%), equivalent to 15-fold of EC_50_ of GT, completely inactivated 1 × 10^8^ PFU of influenza virus. World Health Organization recommends that the concentration of FA or BPL used for virus inactivation should not to exceed 0.1% any time during inactivation. Of note, it has been observed that a fivefold reduction in concentration (<0.02%) often fails to completely inactivate influenza viruses tested (Pawar et al., 2015). Prolonged treatment to ensure complete inactivation and rigorous purification of antigens to remove FA below the threshold level adds further difficulties to the manufacturing process. In contrast, the molar concentration of catechins in 0.1% GT extracts is approximately ∼20-fold lower than the equivalent concentration of FA, allowing higher concentrations of GT, e.g., 0.5%, could be used for complete inactivation of 5 × 10^8^ PFU of influenza viruses.

An essential feature of any inactivating agent is the irreversibility of the reaction to ensure that infectivity is eliminated permanently. GT catechins can form stable complexes with various proteins through autoxidation and subsequent covalent bonding to protein sulfhydryl groups (Ishii et al., 2008; Chen et al., 2011). Worthy of note is that the contrasting antioxidant and prooxidant activities of catechins has been documented (Lambert and Elias, 2010). Catechins initially operate as an antioxidant by scavenging oxygen radicals that in turn activate catechin for subsequent oxidative protein crosslinking. It is not surprising, therefore, that the potency of inactivation was lessened and the kinetics was delayed in the presence of a reducing agent (**Figure 3D**). Moreover, in clear contrast to Tamiflu, a potent inhibitor that is virostatic in nature (Gubareva et al., 2000), the virucidal activity of catechin was irreversible. In all tests examined *in vitro, in ovo*, and *in vivo*, infectivity of GTi viruses was not rescued even after multiple passages. Moreover, attempts to recover infectivity of viruses following prolonged incubation with various biological reducing agents failed, confirming the irreversible nature of the inactivation. Besides crosslinking with surface antigens, it remains an open possibility that catechins also chemically modify the viral membrane lipid components, considering their effect on the morphology of virus particles (Steinmann et al., 2013). The changes in the physical integrity of virions may affect the efficiency of viral and cellular membrane fusion, pivotal for successful infection. The present platform could be widely applied to other viral vaccines as well as to other vaccine production formats. For instance, the present inactivation platform could be used to the manufacture of VLP vaccines (Jeong and Seong, 2017). Most VLP vaccines are produced in insect cells using baculovirus. The purification of VLPs from contaminating baculovirus of a similar size is inherently difficult (Margine et al., 2012), and therefore, the vaccine preparations must be treated with inactivating agents to eliminate residual viral infectivity. Replacing FA with catechins in this inactivation process would preserve the antigenic quality of VLP vaccines for increased vaccine efficacy and safety.

The present report puts forward an alternative and perhaps improved inactivated vaccine platform; however, several issues remain to be addressed. First, while GT catechins in beverages are associated with many health benefits, potential safety issues, especially with injection or intranasal administration of vaccines, remain to be determined. Second, although proof-of-principle is well established with influenza virus and dengue virus, the prototypes of enveloped viruses, whether this approach can be extended to non-enveloped viruses remains to be explored — especially considering that viral inactivation involves not only crosslinking to proteins (especially to thiol residue) but also altering membrane functions. Third, would the present inactivation method and the physical properties of viral antigens be compatible with the pre-existing manufacturing process involving, for instance, sucrose gradient centrifugation and dialysis? Here, consideration should be given to the potential presence of reducing agents during vaccine manufacture that might interfere with inactivation kinetics. Fourth, although GT is equivalent to or even outperforms purified catechins in viral inactivation (Song et al., 2005), the use of extracts of ill-defined composition would pose a regulatory concern. Fifth, could the enhanced immune response to GTi vaccines be translated into a dose-sparing technology? The answer may depend on the threshold concentrations of inactivating agents in the final vaccine products as required for the regulatory approval, which determines the degree of purity and consequently the final yields of a vaccine. The present work is based on crude vaccine preparations, and therefore, a pilot-scale side-by-side comparison of this approach with the current manufacturing process is warranted.

## Conclusion

We present the GT catechin-mediated inactivation of viral infectivity as a novel platform for viral vaccines. With well-known safety and tolerability, GT or its purified components, most notably EGCG, provides favorable alternatives to the design of inactivated viral vaccines against emerging and re-emerging viral diseases.

## Author Contributions

YL, YJ, and BS designed the study, analyzed the data, and wrote the manuscript. YL, YB, YooL, YouL, and JS designed and performed the animal vaccination and challenge experiments and analyzed the antibody responses. YC carried out DENV inactivation by GT. PK performed structural modeling of the binding between EGCG and viral HA. AS, HL, JL, and SY expressed influenza HA and NP proteins using *E. coli* system. YL designed and performed MS experiments. J-MS analyzed the irreversibility of influenza virus inactivation and provided scientific advice to interpret the data related to vaccine efficacy and safety.

## Conflict of Interest Statement

The authors declare that the research was conducted in the absence of any commercial or financial relationships that could be construed as a potential conflict of interest.
