# Supplementary material for: Green Tea Catechin-Inactivated Viral Vaccine Platform
Source: Front Microbiol. 2017 Dec 12;8:2469. doi: 10.3389/fmicb.2017.02469 (PMC5732980; doi:10.3389/fmicb.2017.02469)
Supplement: Supplementary file 1 [file Presentation_1.PDF]

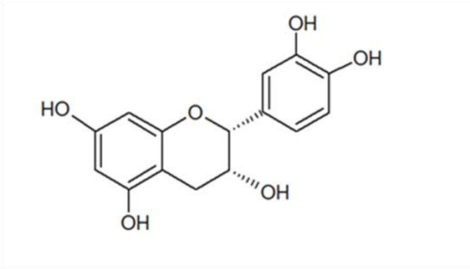

Epicatechin (EC)

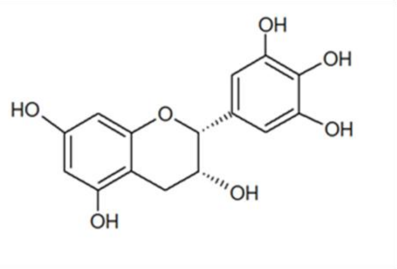

Epigallocatechin (EGC)

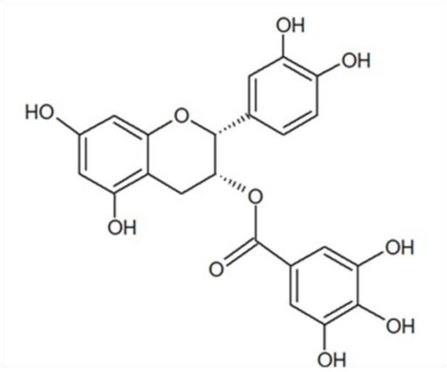

Epicatechin-3-gallate (ECG)

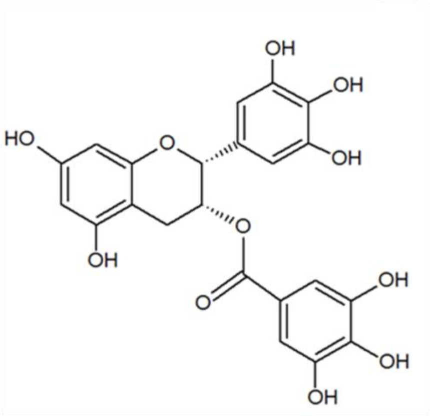

Epigallocatechin-3-gallate (EGCG)

Supplementary Figure S1. Chemical structures of green tea catechins. Structures of epicatechin (EC), epigallocatechin (EGC), epicatechin-3-gallate (ECG), and epigallocatechin-3-gallate (EGCG) are shown.

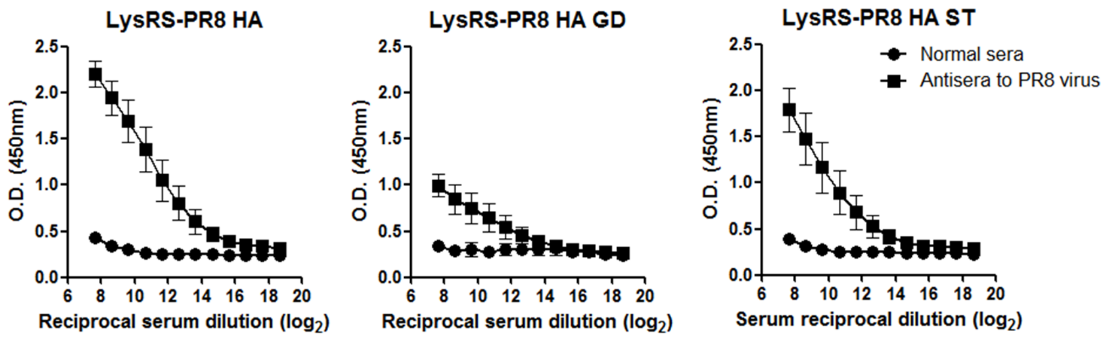

Supplementary Figure S2. E.coli-expressed HA proteins strongly bound to antisera to PR8 virus. 96-well plates were coated with E.coli-expressed PR8 HA full-length, globular domain, and stalk proteins, and two-fold serial dilutions of mice normal sera (n = 3) or antisera to PR8 virus (n = 5) were added to the wells to measure specific IgG antibody binding to each protein by ELISA. Data are the mean of each cohort.

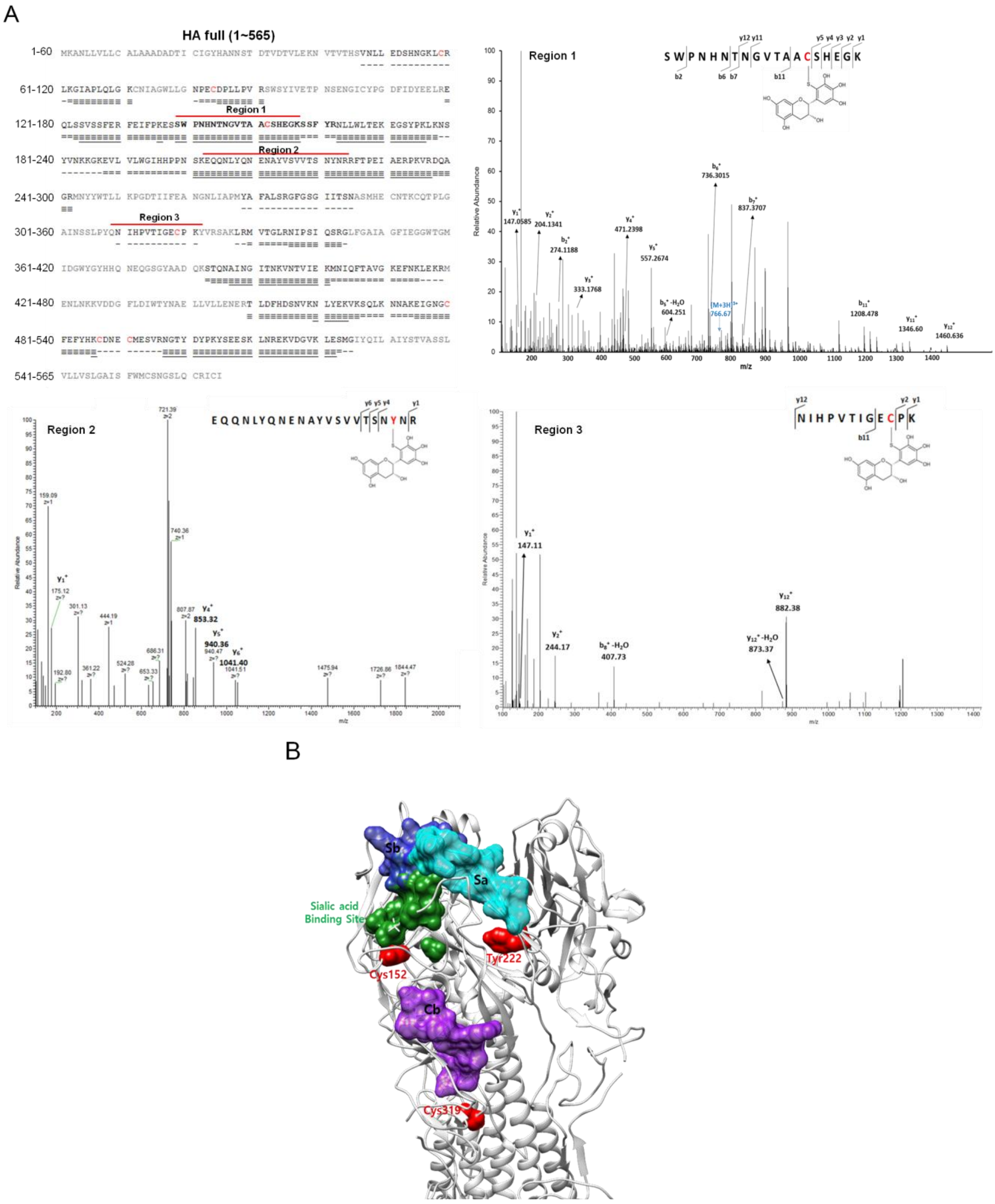

Supplementary Figure S3. Mass analysis of influenza HA proteins treated with EGCG. (**A**) Mass analysis of the HA full-length proteins treated with EGCG. The HA proteins were incubated with 10 μM of EGCG for 2 h at RT before mass spectrometry. Red lines represent EGCG binding regions identified in LC-MS/MS. (**B**) EGCG binding sites on influenza HA protein. The 3D structure of PR8 virus HA protein was obtained from the Protein Data Bank (PDB 1RVX). Three antigenic sites (Sa, Sb, and Cb) and receptor (sialic acid) binding site were visualized by surface model. Three amino acids that were shown to be conjugated with EGCG in mass spectrometry are colored in red (Cys_{152}, Cys_{319}, and Tyr_{222}). Visualization was carried out using UCSF-CHIMERA (Resource for Biocomputing, Visualization, and Informatics. Ver 1.11).

A

A. Sa: Biotin-GGEKEGSYPKLKNS

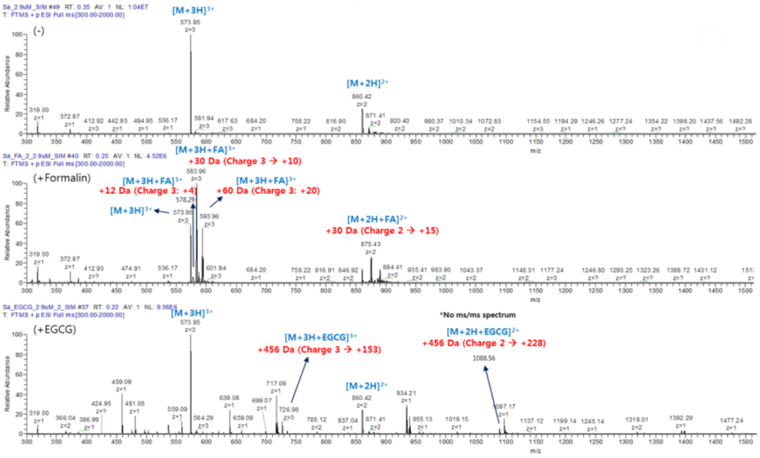

A. Sa + FA

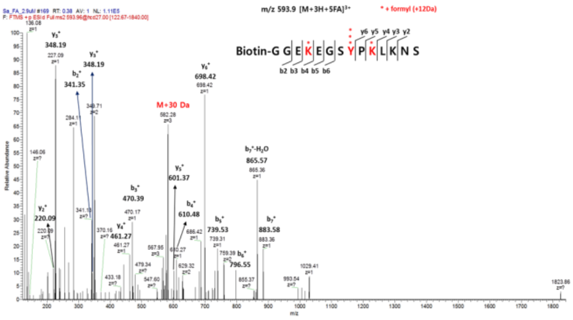

A. Sa + EGCG

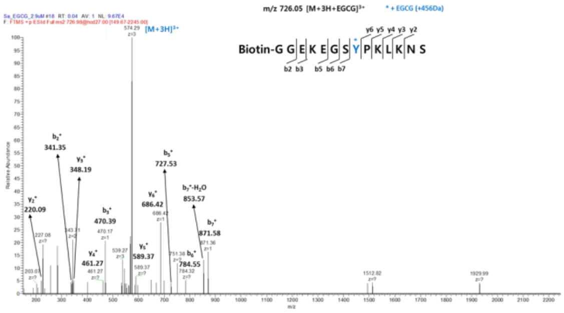

B

B. Sb: Biotin-GGNSKEQQNLYQNE

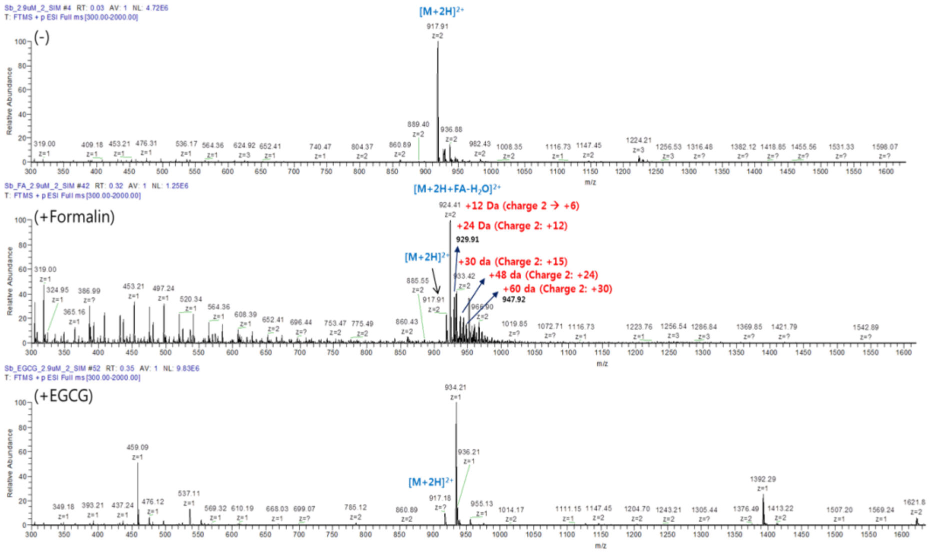

B. Sb + FA

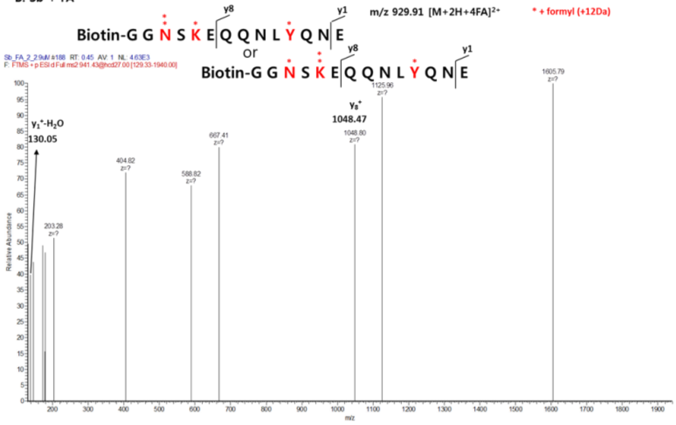

C

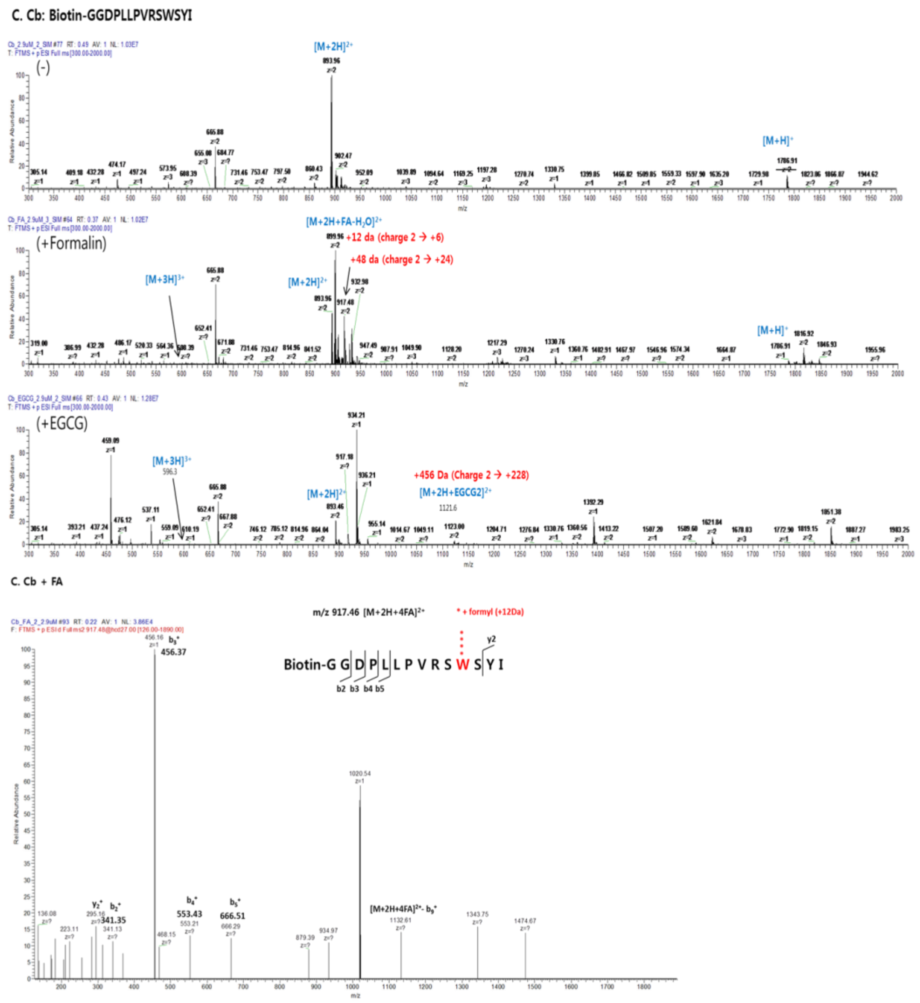

Supplementary Figure S4. Mass analysis of influenza HA epitopes treated with FA or EGCG. 750 μM of each peptide were mixed with 0.15% of FA or EGCG and incubated for 24 h at 37 ℃. To determine EGCG binding residues, mass analysis of FA- or EGCG-treated Sa (156–167) (**A**), Sb (187–198) (**B**), and Cb (77–82) (**C**) peptides were performed by direct infusion-SIM and direct infusion-MS/MS methods
